# Supplementary material for: Heterogeneity of hepatocyte dynamics restores liver architecture after chemical, physical or viral damage
Source: Nat Commun. 2024 Feb 10;15:1247. doi: 10.1038/s41467-024-45439-0 (PMC10858916; doi:10.1038/s41467-024-45439-0)
Supplement: Supplementary file 1 — Supplementary Information [file 41467_2024_45439_MOESM1_ESM.pdf]

Heterogeneity of hepatocyte dynamics restores liver architecture after chemical, physical or viral damage

Inmaculada Ruz-Maldonado[1, 2, 3, 4, 5], John T. Gonzalez[1, 2, 3], Hanming Zhang[1, 2, 3, 5], Jonathan Sun[1, 2, 3, 5], Alicia Bort[1, 2, 3, 5], Inamul Kabir[6, 7], Richard G Kibbey[4], Yajaira Suárez[1, 2, 3, 5], Daniel M. Greif[6, 7], Carlos Fernández-Hernando[1, 2, 3, 5, *]

[1]Vascular Biology and Therapeutics Program, Yale University School of Medicine, New Haven, CT, 06520, USA.

[2]Department of Comparative Medicine, Yale University School of Medicine, New Haven, CT, 06520, USA.

[3]Yale Center of Molecular and Systems Metabolism, Yale University School of Medicine, New Haven, CT, 06520, USA.

[4]Departments of Internal Medicine (Endocrinology) and Cellular & Molecular Physiology, Yale University, New Haven, CT, USA.

[5]Department of Pathology, Yale University School of Medicine, New Haven, CT, 06520, USA.

[6]Yale Cardiovascular Research Center, Section of Cardiovascular Medicine, Department of Internal Medicine, Yale University School of Medicine, New Haven, CT 06511 USA

[7]Department of Genetics, Yale University School of Medicine, New Haven, CT 06511 USA

SUPPLEMENTAL INFORMATION

Supplementary Figure 1

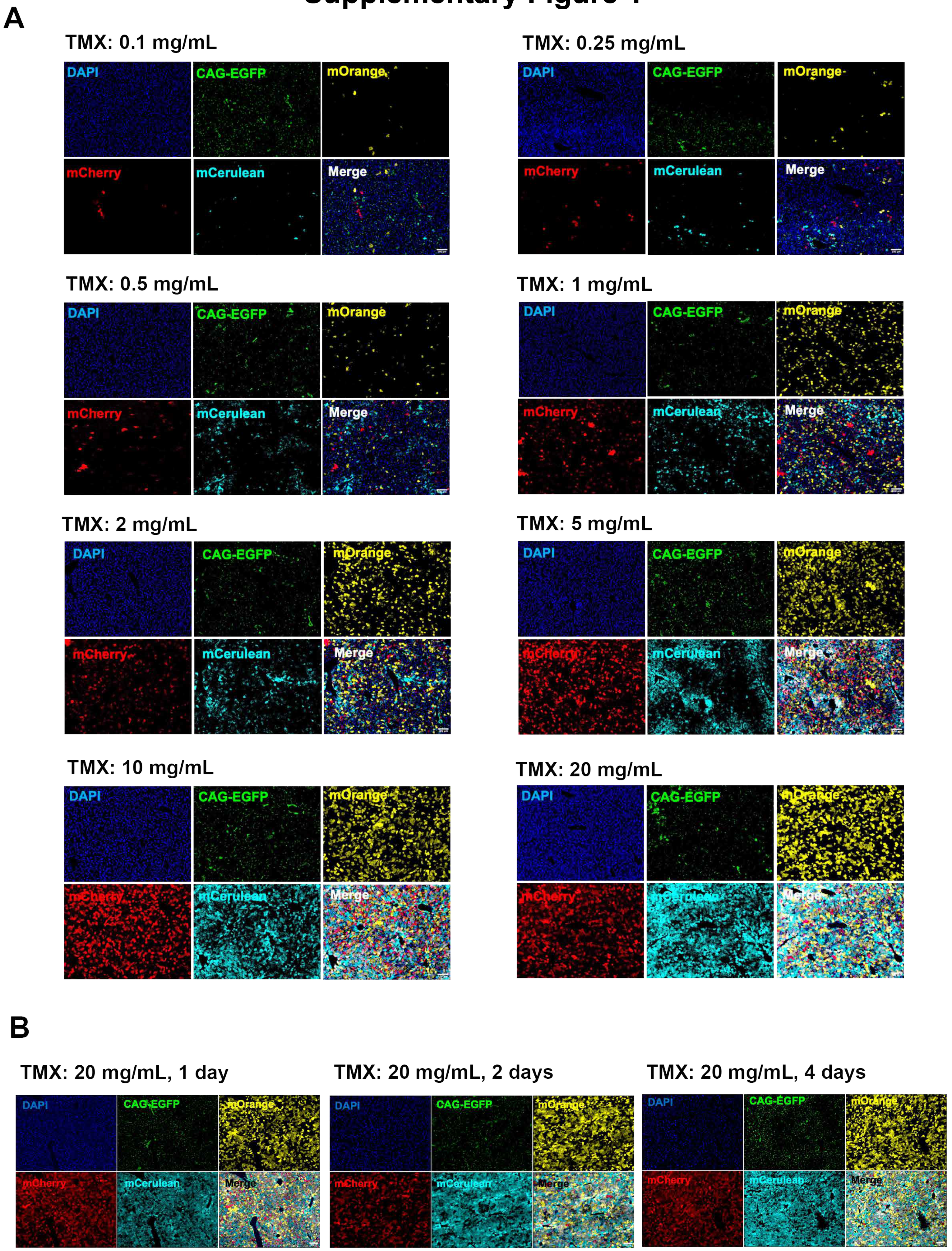

Supplementary Figure 1. Optimization of the tamoxifen dose for Cre recombinase activation in livers from *Alb-CreERT2 Rosa26^{rbw}* mice.

(A) Tamoxifen dose-response test in liver sections from 11-week-old female Alb-CreERT2 Rosa26^{rbw} mice. Representative images of liver sections from Alb-CreERT2 Rosa26^{rbw} mice after 1 week of 3 intraperitoneal injections of TMX during 3 consecutive days (100 µL per day) at 0.1 mg/mL, 0.25 mg/mL, 0.5 mg/mL, 1 mg/mL, 0.2 mg/mL, 0.5 mg/mL, 10 mg/mL and 20 mg/mL. Note that the activation of fluorophores (CAG-EGFP, mOrange, mCherry and mCerulean) is proportional to TMX dose, with the presence of more labeled cells as TMX concentration increases. TMX: tamoxifen. N= 3 mice. Scale bars, 100 µm.

(B) Tamoxifen time-course test in liver sections from 11-week-old female Alb-CreERT2 Rosa26^{rbw} mice. Representative images of liver sections from Alb-CreERT2 Rosa26^{rbw} mice after 1 week of 1, 2 or 4 intraperitoneal injections of 20 mg/mL TMX during 1, 2 or 4 consecutive days respectively (100 µL per day). N= 3 mice. Scale bars, 100 µm.

Supplementary Figure 2

A

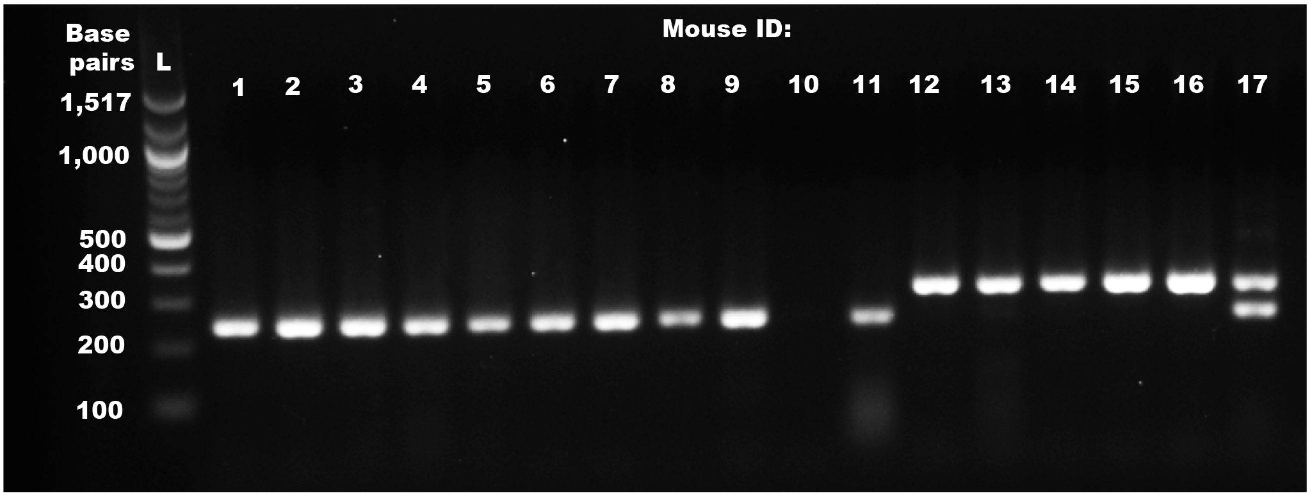

- Mice 1-9 and 11: Homozygous Rosa26^{rbw}

- Mice 12-16: Homozygous WT

- Mouse 17: Heterozygous Rosa26^{rbw}-WT

B

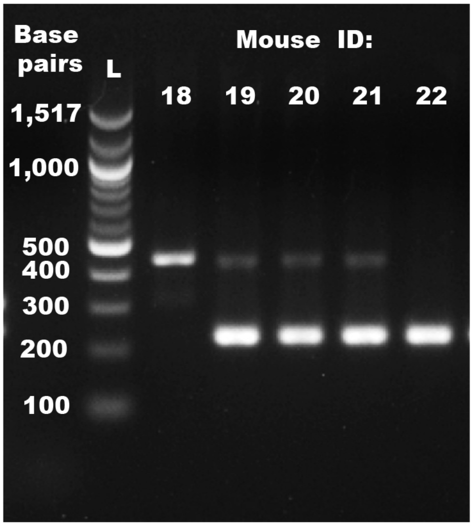

- Mouse 18: Homozygous Alb-CreERT2

- Mice 19-21: Heterozygous Alb-CreERT2-WT

- Mouse 22: Homozygous WT

Supplementary Figure 2. *Rosa26^{rbw}* and *Alb-CreERT2* mouse genotyping.

(A) Representative agarose gel electrophoresis image of PCR products from homozygous and heterozygous Rosa26^{rbw} mice. L: 100 bp DNA ladder.

(B) Representative agarose gel electrophoresis image of PCR products from homozygous and heterozygous Alb-CreERT2 mice L: 100 bp DNA ladder.

Supplementary Figure 3

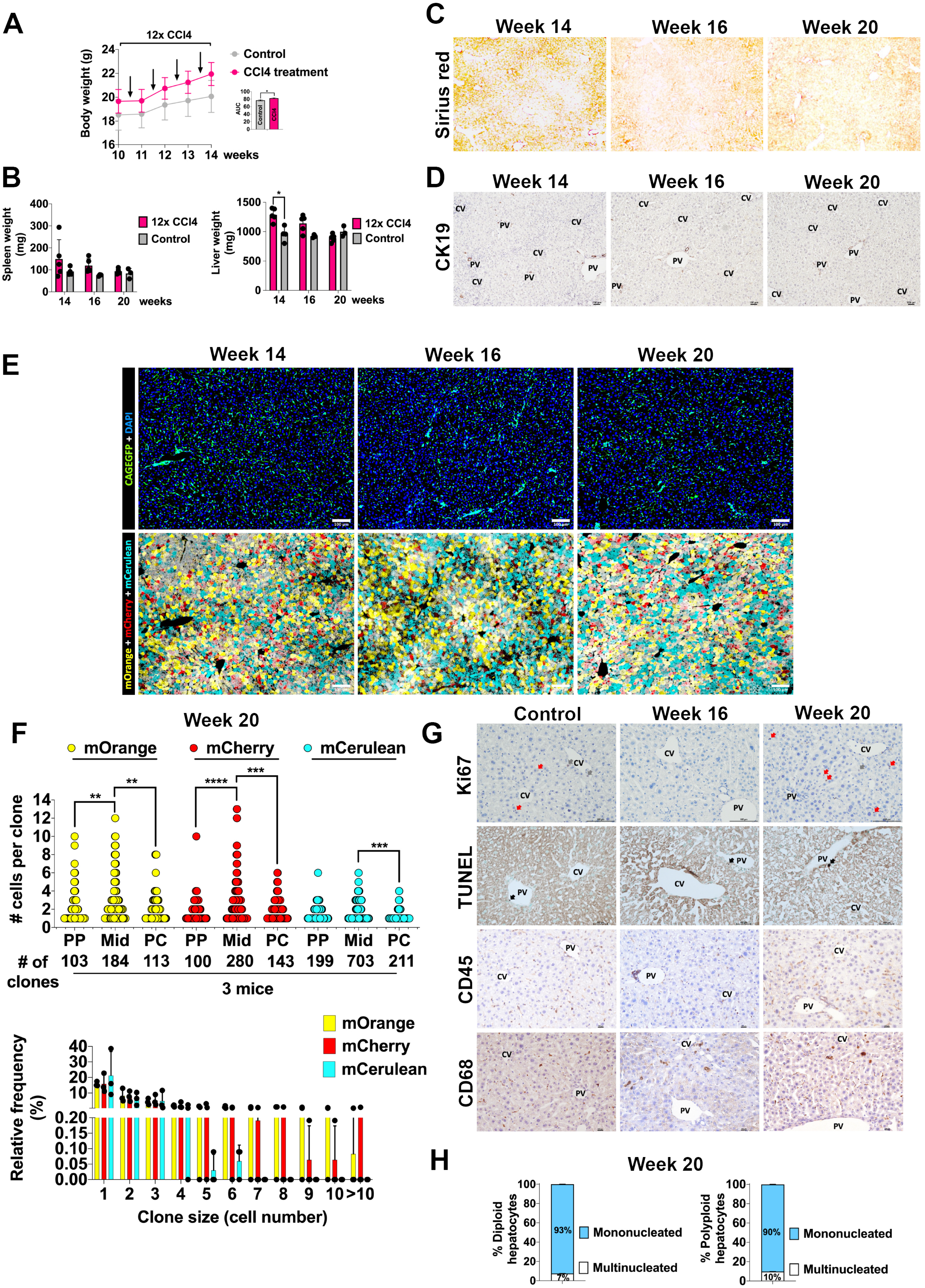

Supplementary Figure 3. **Clonal expansion of hepatocytes after CCl4-induced chronic liver injury**.

(A) Weekly body weights (g) and area under the curve (AUC) quantification from female Alb-CreERT2 Rosa26^{rbw} mice that received 12 intraperitoneal injections of CCl4 or vehicle (control mice) every 3 days during 4 weeks. Arrows indicate the administration of 3 doses of CCl4 per week. 9 and 12 total mice were used for control and CCl4 treatment respectively. Data are presented as mean values +/- SEM. Unpaired t test; *p < 0.05.

(B) Spleen and liver weights (mg) from female Alb-CreERT2 Rosa26^{rbw} mice euthanized at 14, 16 and 20 weeks of age, after receiving 12 injections of CCl4 or vehicle during 4 weeks starting the treatments at week 10 of age. 3-4 control and 4-5 CCl4 treated mice were used per time point. Two-way ANOVA, Sidak's multiple comparisons post-test. Data are presented as mean values +/- SEM.*P< 0.05.

(C) Sirius red staining in liver sections from female Alb-CreERT2 Rosa26^{rbw} mice that were treated with vehicle for 4 weeks at 10 weeks of age and were euthanized at week 14, 16 and 20 of age.

(D) CK19 IHC in liver sections from female Alb-CreERT2 Rosa26^{rbw} mice administered with additional doses of CCl4 for 4 weeks at 10 weeks of age and euthanized at week 14, 16 and 20 of age. CV: central vein; PV: portal vein. Scale bars, 100 µm.

(E) Liver sections from female Alb-CreERT2 Rosa26^{rbw} mice at 14, 16 and 20 weeks of age after receiving additional doses of vehicle. Top panels show CAG-EGFP staining (green) expressed by non-hepatocyte cells and DAPI staining, that shows the nuclei of total cells found in the field of view 10x. Bottom panels indicate the Rainbow mOrange (yelow), mCherry (red) and mCerulean (light blue) fluorophores expressed by hepatocytes. Scale bars, 100 µm.

(F) Number, size and relative frequency of hepatocyte clones in 20-week-old female Alb-CreERT2 Rosa26^{rbw} mice treated with vehicle. *Top graph:* quantification of number of cells per clone (y- axis) and number of clones (x-axis) per area of the liver lobule and per Rainbow fluorophore of liver sections from Alb-CreERT2 Rosa26^{rbw} mice treated with vehicle and euthanized at 20 weeks of age. Data are presented as mean values. 27 liver lobule areas were analyzed from 3 mice per time- point (3-5 photos per mouse). One-way ANOVA, Tukey's multiple comparisons post-test; ****p < 0.0001; ***p < 0.001; **p < 0.01. PP: periportal hepatocytes; Mid: midlobular hepatocytes; PC: pericentral hepatocytes. *Bottom graph*: relative frequency in % of each clone size per area of the liver lobule and per Rainbow fluorophore from female Alb-CreERT2 Rosa26^{rbw} mice at week 20 of age, treated with vehicle. 27 liver lobule areas were analyzed from 3 mice per time-point (3-5 photos per mouse). Data are presented as mean values +/- SEM.

(G)Ki67, CD45 and CD68 IHC and TUNEL assay in liver sections from Alb-CreERT2 Rosa26^{rbw} mice treated with additional doses of vehicle (control) or CCl4 at the recovery periods, week 16 and 20 of age. Grey arrows show proliferating Ki67 hepatocytes whereas red arrows show proliferating Ki67 non-hepatocyte cells. Black arrows indicate apoptotic cells. Scale bars, 100 µm.

(H) Hepatocyte ploidy in % in the liver lobule of 20-week-old female Alb-CreERT2 Rosa26^{rbw} mice after 6 weeks of recovery period, post-CCl4-induced chronic injury. *Left graph:* % of diploid hepatocytes (hepatocytes that express only 1 fluorophore) being mononucleated or multinucleated. *Right graph*: % of polyploid hepatocytes (hepatocytes that express more than 1 fluorophore) being mononucleated or multinucleated. 5 photos were analyzed per mouse for a total of 3 mice. Data are presented as mean values +/- SEM.

Source data are provided as a Source Data file.

Supplementary Figure 4

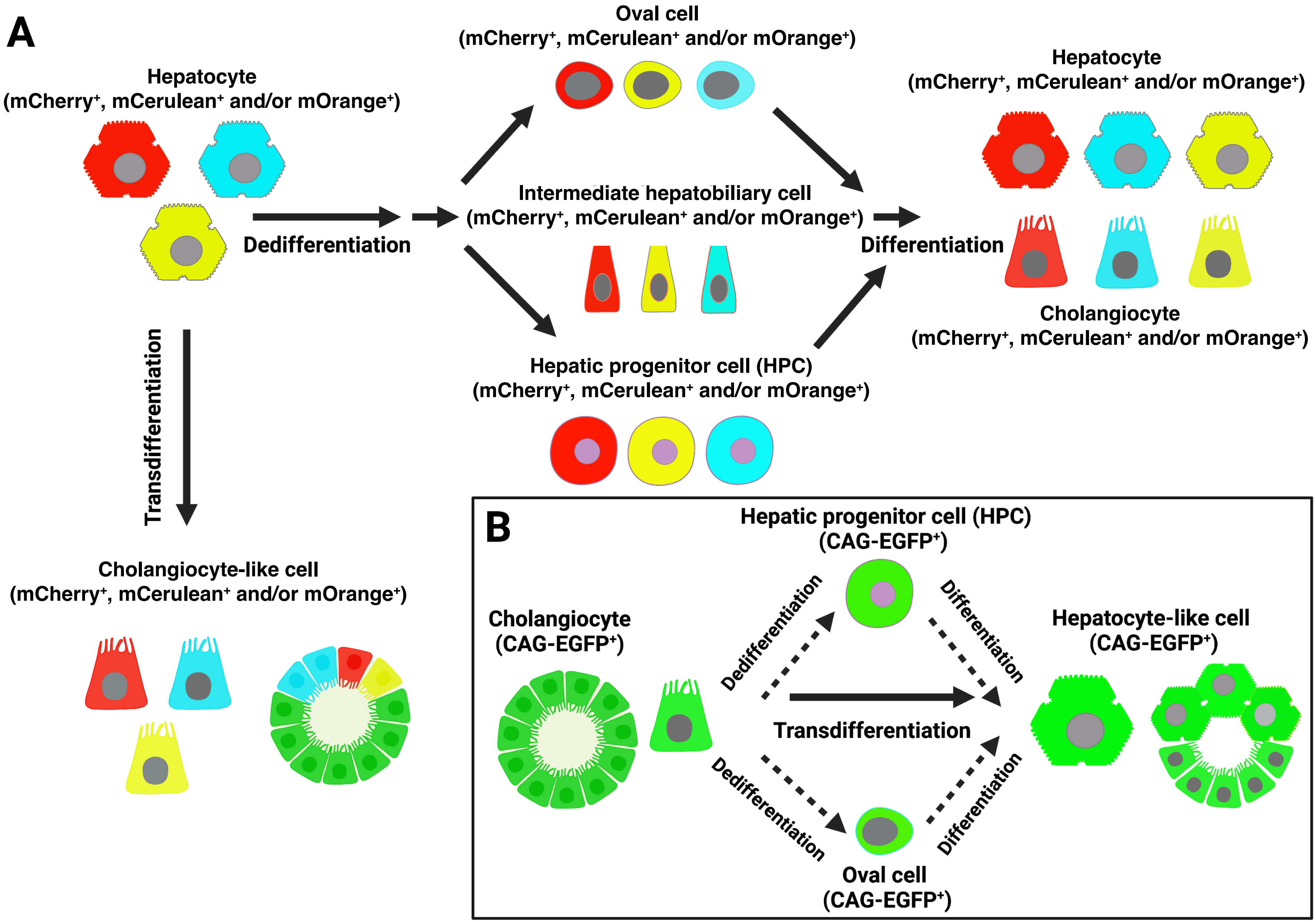

Supplementary Figure 4. Schematic of multicolor tracking of potential transdifferentiation mechanisms and intermediate cell types between hepatocytes and cholangiocytes in the liver of *Alb-CreERT2 Rosa26^{rbw}* mice.
(A) Transdifferentiation of hepatocytes to cholangiocytes.
(B) Transdifferentiation of cholangiocytes to hepatocytes.
Created with BioRender.com.

Supplementary Figure 5

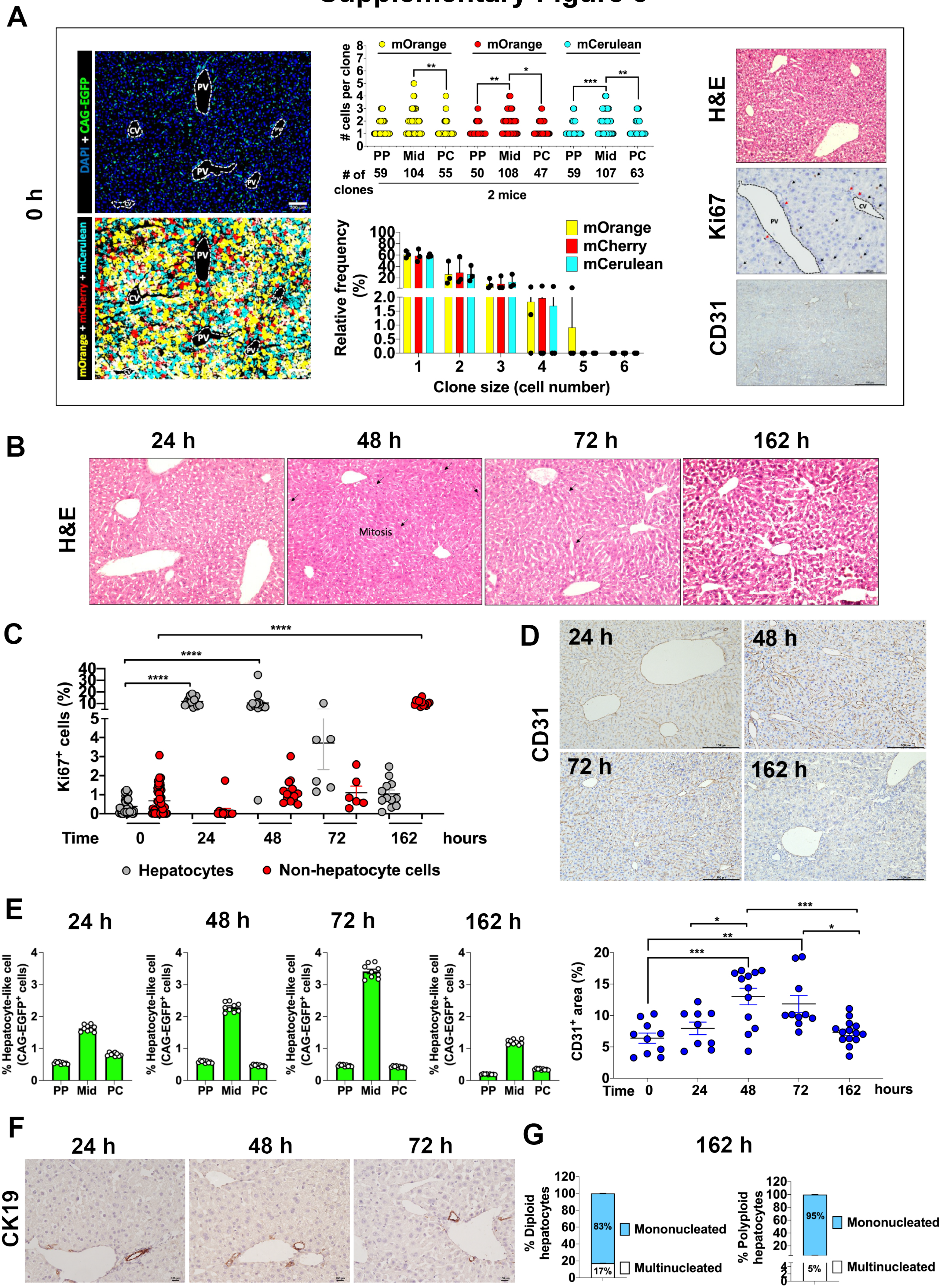

Supplementary Figure 5. **Liver regeneration dynamics after 2/3 partial hepatectomy.**
(A) Histological analysis of liver lobes from Alb-CreERT2 Rosa26^{rbw} female mice used as hour 0 in the 2/3 partial hepatectomy (PHx) procedure. *Left:* Images of Rainbow fluorophore staining; CAG-EGFP is expressed by non-hepatocyte cells. DAPI staining shows the nuclei of total cells found in the field of view 10x. mOrange (yelow), mCherry (red) and mCerulean (light blue) fluorophores are expressed by hepatocytes. PV: periportal vein; PC: pericentral vein. Scale bars, 100 μm. *Middle*: number, size and relative frequency of hepatocyte clones in 10-week- old female Alb-CreERT2 Rosa26^{rbw} mice at time 0 hours before 2/3 PHx. *Top middle graph*: quantification of number of cells per clone (y-axis) and number of clones (x-axis) per area of the liver lobule and per Rainbow fluorophore. (11 liver lobule areas were analyzed from 2 mice per time-point (3-4 photos per mouse). Data are presented as mean values. One-way ANOVA, Tukey's multiple comparisons post-test; ***$p < 0.001$; **$p < 0.01$; *$p < 0.05$. PP: periportal hepatocytes; Mid: midlobular hepatocytes; PC: pericentral hepatocytes. *Bottom middle graph*: relative frequency in % of each clone size per area of the liver lobule and per Rainbow fluorophore. Data are presented as mean values +/- SEM. 11 liver lobule areas were analyzed from 3 mice per time-point (3-4 photos per mouse). *Right*: H&E, Ki67 and CD31 staining. Scale bars, 100 μm.
(B) H&E staining of liver sections from Alb-CreERT2 Rosa26^{rbw} female mice 24, 48, 72 and 162 h after 2/3 PHx. Arrows indicate mitosis.
(C) Quantification of total Ki67 positive cells (hepatocytes and non-hepatocyte cells) in % per field of view (10x) of liver sections from Alb-CreERT2 Rosa26^{rbw} mice at 0, 24, 48, 72 and 162 h after 2/3 PHx. 10-27 liver lobule areas were analyzed from 2 mice per time point. Data are presented as mean values +/- SEM. One-way ANOVA, Tukey's multiple comparisons post-test; **** $p < 0.001$.
(D) Images and quantification of CD31 IHC in liver sections from Alb-CreERT2 Rosa26^{rbw} female mice 24, 48, 72 and 162 h after 2/3 PHx. 9-14 photos were analyzed from 2 mice. Data are presented as mean values +/- SEM. One-way ANOVA, Tukey's multiple comparisons post-test; ***$p < 0.001$; **$p < 0.01$; *$p < 0.05$. Scale bars, 100 μm.
(E) Quantification of % CAG-EGFP positive hepatocyte cells-like per area of the liver lobule (per field of view 10x) from Alb-CreERT2 Rosa26^{rbw} mice subjected to 2/3 PHx after 24, 48, 72 and 162 h. Data are presented as mean values +/- SEM.
(F) CK19 IHC in liver sections from Alb-CreERT2 Rosa26^{rbw} female mice 24, 48 and 72 h after 2/3 PHx. Scale bars, 100 μm.
(G) Hepatocyte ploidy in % in the liver lobule of Alb-CreERT2 Rosa26^{rbw} female mice 162 h after 2/3 PHx. *Left graph*: % of diploid hepatocytes (hepatocytes that express only 1 fluorophore) being mononucleated or multinucleated. *Right graph*: % of polyploid hepatocytes (hepatocytes that express more than 1 fluorophore) being mononucleated or multinucleated. Data are presented as mean values +/- SEM. 5 photos were analyzed per mouse. N= 2 mice. Source data are provided as a Source Data file.

Supplementary Figure 6

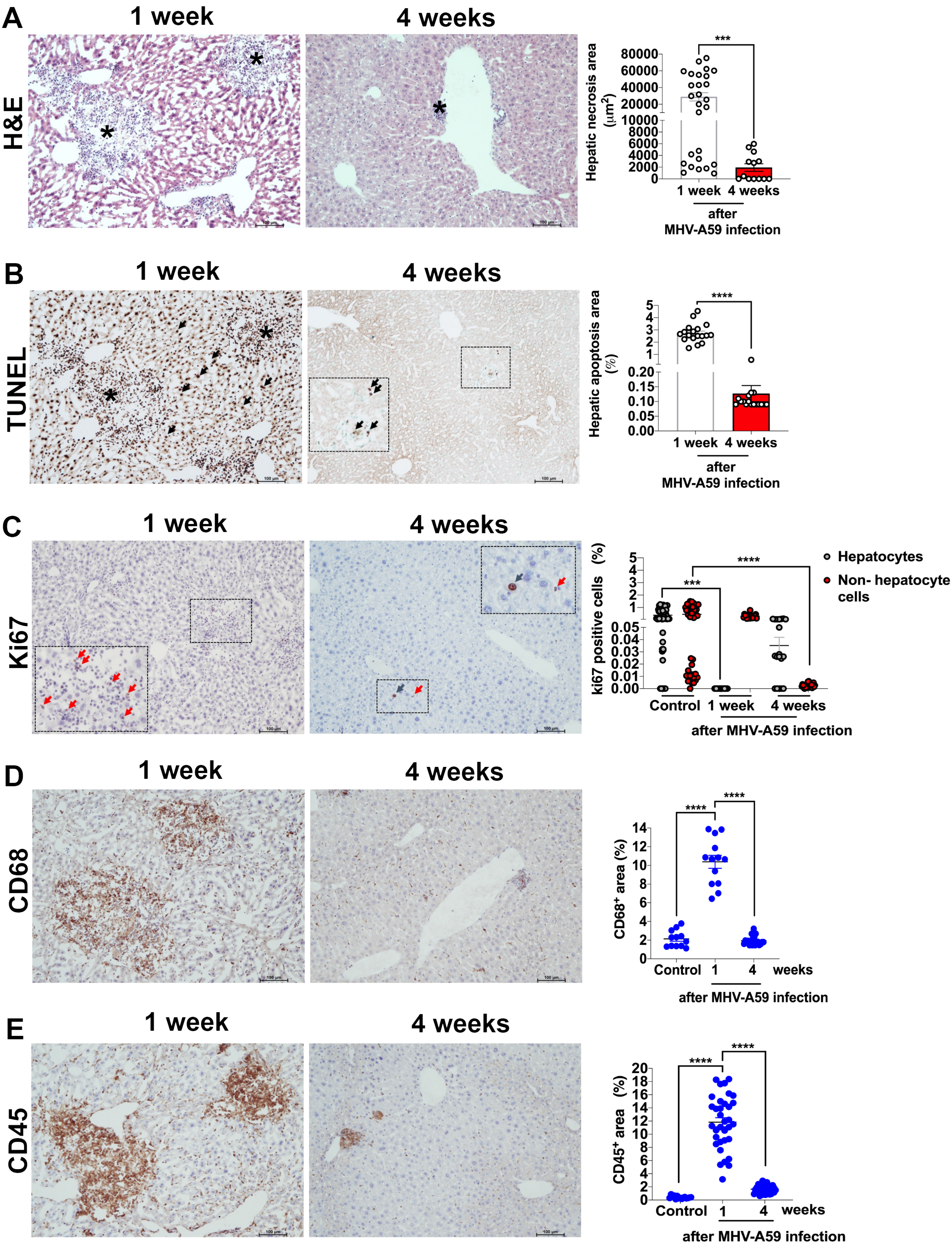

Supplementary Figure 6. Liver damage and repair after MHV-A95 coronavirus infection.
(A) Images and necrosis quantification of H&E staining in liver sections from Alb- CreERT2 Rosa26^{rbw} male mice at 1 and 4 weeks post MHV-A95 infection. Asterisks indicate immune infiltrate. Scale bars, 100 µm. Data are presented as mean values +/- SEM. 4-9 photos were analyzed from 2 mice. Unpaired t test; ***p < 0.001.
(B) Images and quantification of TUNEL staining in liver sections from Alb- CreERT2 Rosa26^{rbw} male mice at 1 and 4 weeks post MHV-A95 infection. Asterisks indicate immune infiltrate. Arrows show apoptotic cells. Scale bars, 100 µm. Data are presented as mean values +/- SEM. 6-16 photos were analyzed from 2 mice. Unpaired t test; ****p < 0.0001.
(C) Images and quantification of Ki67 IHC in liver sections from Alb-CreERT2 Rosa26^{rbw} male mice at 1 and 4 weeks post MHV-A95 infection. Red arrows show proliferating non- hepatocyte cells. Grey arrows indicate proliferating hepatocytes. Scale bars, 100 µm. Data are presented as mean values +/- SEM . 20-25 photos were analyzed from 2 mice. One-way ANOVA, Tukey's multiple comparisons post-test; **** p< 0.001; ***p <0.001.
(D) Images and quantification of CD68 IHC in liver sections from Alb-CreERT2 Rosa26^{rbw} male mice at 1 and 4 weeks post MHV-A95 infection or vehicle treated (control mice). Scale bars, 100 µm. Data are presented as mean values +/- SEM. 12-21 photos were analyzed from 2 mice. One-way ANOVA, Tukey's multiple comparisons post-test; **** p < 0.0001.
(E) Images and quantification of CD45 IHC in liver sections from Alb-CreERT2 Rosa26^{rbw} male mice at 1 and 4 weeks post MHV-A95 infection or vehicle treated (control mice). Scale bars, 100 µm. Data are presented as mean values +/- SEM. 15-16 photos were analyzed from 2 mice. One-way ANOVA, Tukey's multiple comparisons post-test; **** p< 0.0001.
Source data are provided as a Source Data file.

Supplementary Figure 7

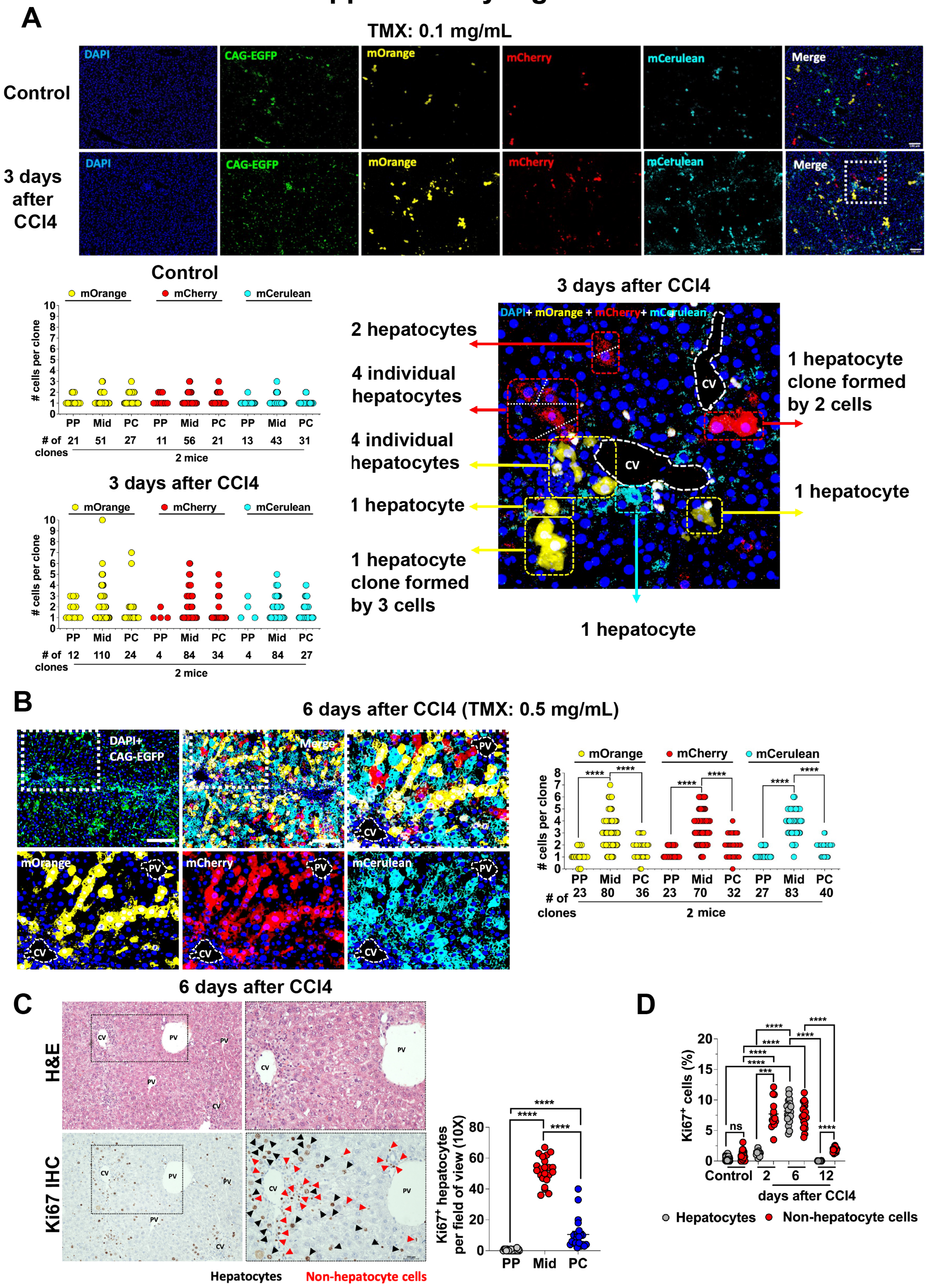

Supplementary Figure 7. Visualization of hepatocyte clones at low TMX concentrations and hepatocyte clonal quantification and proliferation at day 3 and 6 after CCl4 acute treatment.
(A) Detailed images of hepatocyte clone generation and its quantification after 3 days of acute CCl4 treatment or vehicle (control) in liver sections from female Alb-CreERT2 Rosa26^{rbw} mice after 1 week of 3 intraperitoneal injections of TMX during 3 consecutive days (100 µL per day) at 0.1 mg/mL. 2 mice per control and per CCl4 treatment were used. Data are presented as mean values. CV: central vein; PP: periportal hepatocytes; Mid: midlobular hepatocytes; PC: pericentral hepatocytes. Scale bars, 100 µm.
(B) Detailed images of hepatocyte clone generation and its quantification after 6 days of acute CCl4 treatment in liver sections from female Alb-CreERT2 Rosa26^{rbw} mice after 1 week of 3 intraperitoneal injections of TMX during 3 consecutive days (100 µL per day) at 0.5 mg/mL. Data are presented as mean values. N= 2 mice. One-way ANOVA, Tukey's multiple comparisons post-test; **** $p < 0.001$. Scale bars, 100 µm.
(C) H&E and Ki67 staining and quantification of hepatocyte proliferation in liver sections from Alb-CreERT2 Rosa26^{rbw} mice after 6 days of acute treatment with CCl4. Data are presented as mean values +/- SEM. One-way ANOVA, Tukey's multiple comparisons post-test; **** $p < 0.001$. N= 3 mice.
(D) Proliferation rate by Ki67 staining expressed in hepatocytes and non-hepatocyte cells in liver sections from Alb-CreERT2 Rosa26^{rbw} control mice and mice treated with CCl4 after 2, 6 and 12 days. Data are presented as mean values +/- SEM. One-way ANOVA, Tukey's multiple comparisons post-test; *** $p < 0.01$; **** $p < 0.001$. N= 3 mice per group.
Source data are provided as a Source Data file.

Supplementary Figure 8

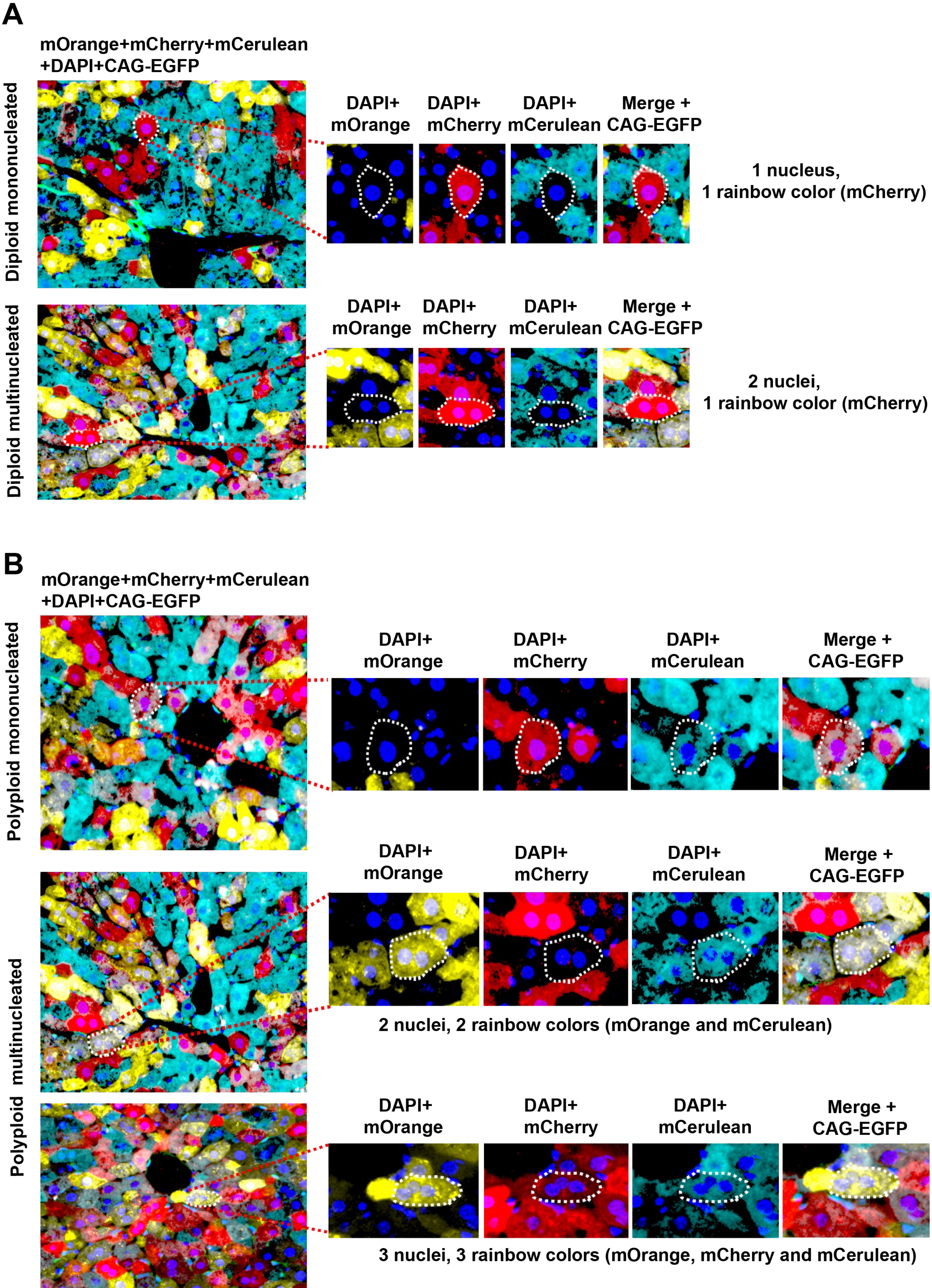

Supplementary Figure 8. Hepatocyte ploidy visualization.
(A) Detailed images of diploid hepatocytes mononucleated and multinucleated. N=3 mice.
(B) Detailed images of polyploid hepatocytes mononucleated and multinucleated. N=3 mice.

Supplementary Figure 9

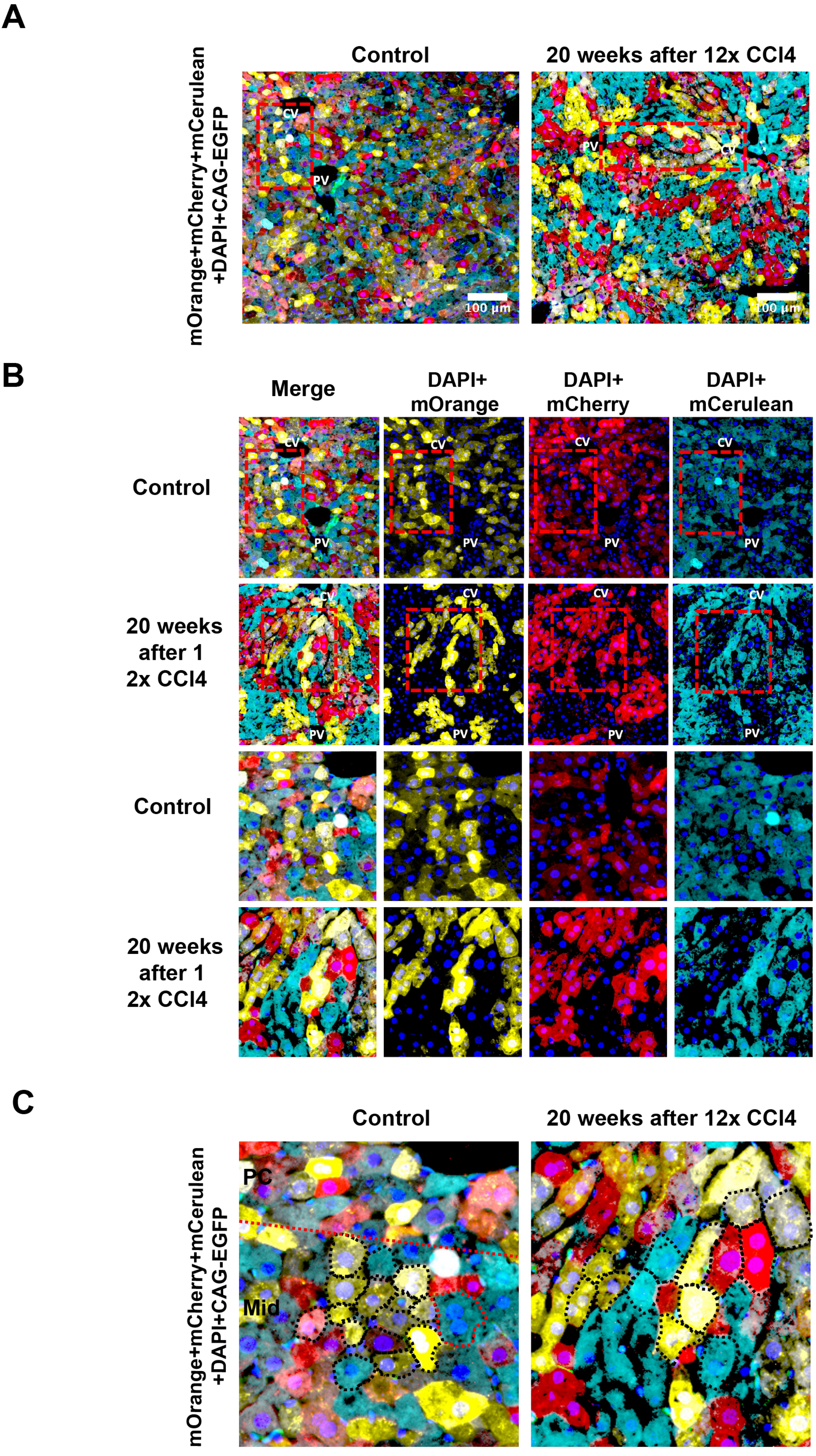

Supplementary Figure 9. Hepatocyte hypertrophy visualization after chronic treatment with CCl4.

(A) Liver sections from Alb-CreERT2 Rosa26^{rbw} mice after 20 weeks of CCl4 chronic treatment or vehicle (control). CV: central vein; PV: portal vein. N= 3 mice.

(B) Detailed views of A showing a liver lobule (the distance comprised between a periportal and a pericentral vein) from livers from control and CCl4 mice treated mice. N= 3 mice.

(C) Detailed views of B indicating hepatocyte hypertrophy in midlobular hepatocytes from liver sections of Alb-CreERT2 Rosa26^{rbw} mice after chronic CCl4 treatment compared to control mice. N=3 mice.

Supplementary Figure 10

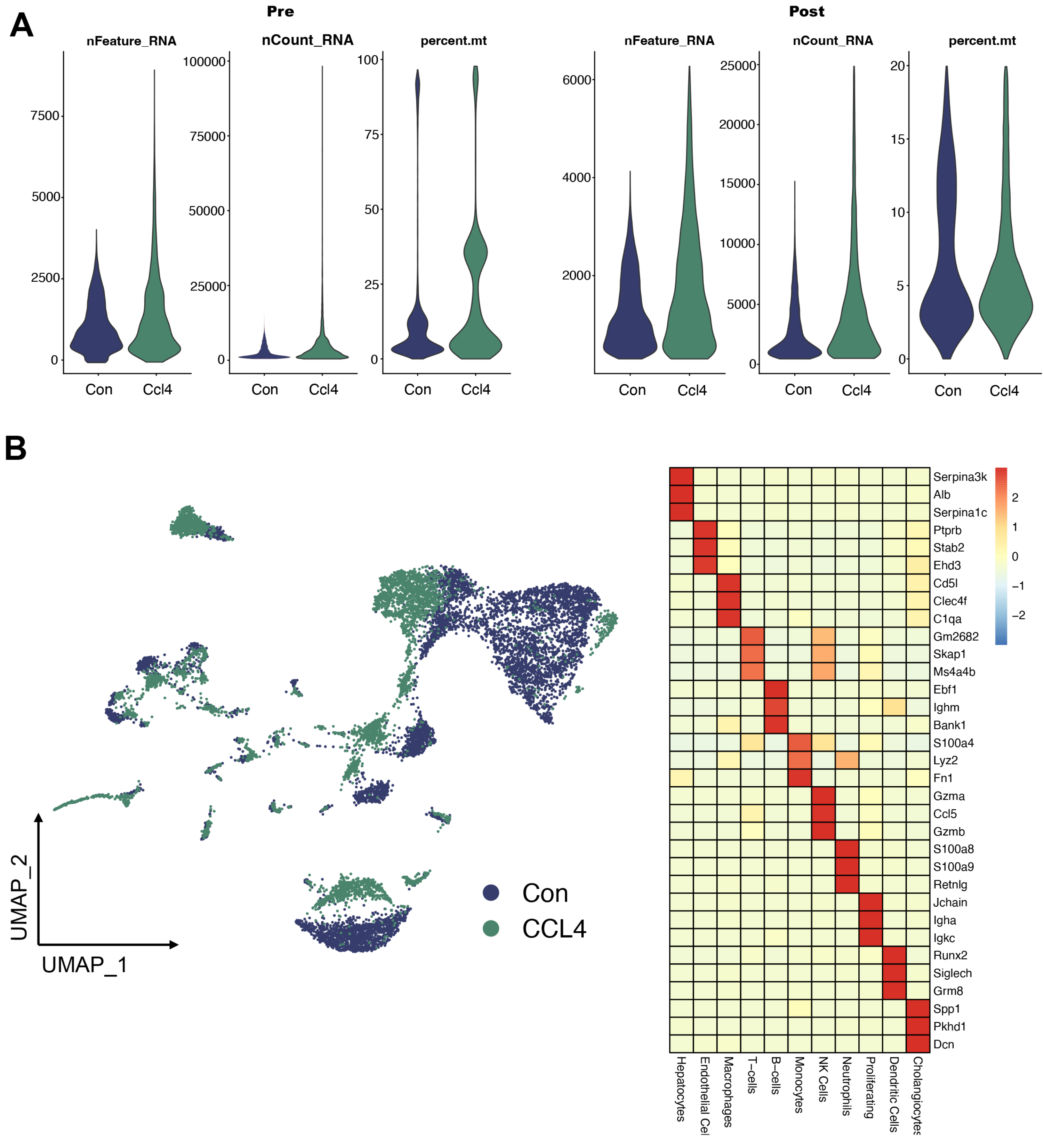

Supplementary Figure 10. scRNA-seq data from liver preparations isolated from Alb-CreERT2 Rosa 26^{rbw} mice after 6 days of being treated with a single dose of vehicle (control) or CCl4 (CCl4).

(A) scRNA-seq data quality control pre and post exclusion of non-viable and low-quality cells from Alb-CreERT2 Rosa 26^{rbw} mice after 6 days of being treated with a single dose of vehicle (control) or CCl4 (CCl4). Violin plots showing the number of detected genes (nFeature_RNA), mRNA counts (nCount-RNA) and percentage of mitochondrial genes (percent_mt) across all samples). N=2 Alb-CreERT2 Rosa26^{rbw} mice per group.

(B) UMAP clustering of single cell data with Seurat from liver preparations isolated from Alb-CreERT2 Rosa 26^{rbw} mice after 6 days of being treated with a single dose of vehicle (control) or CCl4 (CCl4). Liver cells were clustered in 2 dimensions using the UMAP dimensionality reduction technique and annotated by experimental identity. Top 3 differentially expressed marker genes are shown for each cell cluster. N=2 Alb-CreERT2 Rosa26^{rbw} mice per group.

Supplementary Figure 11

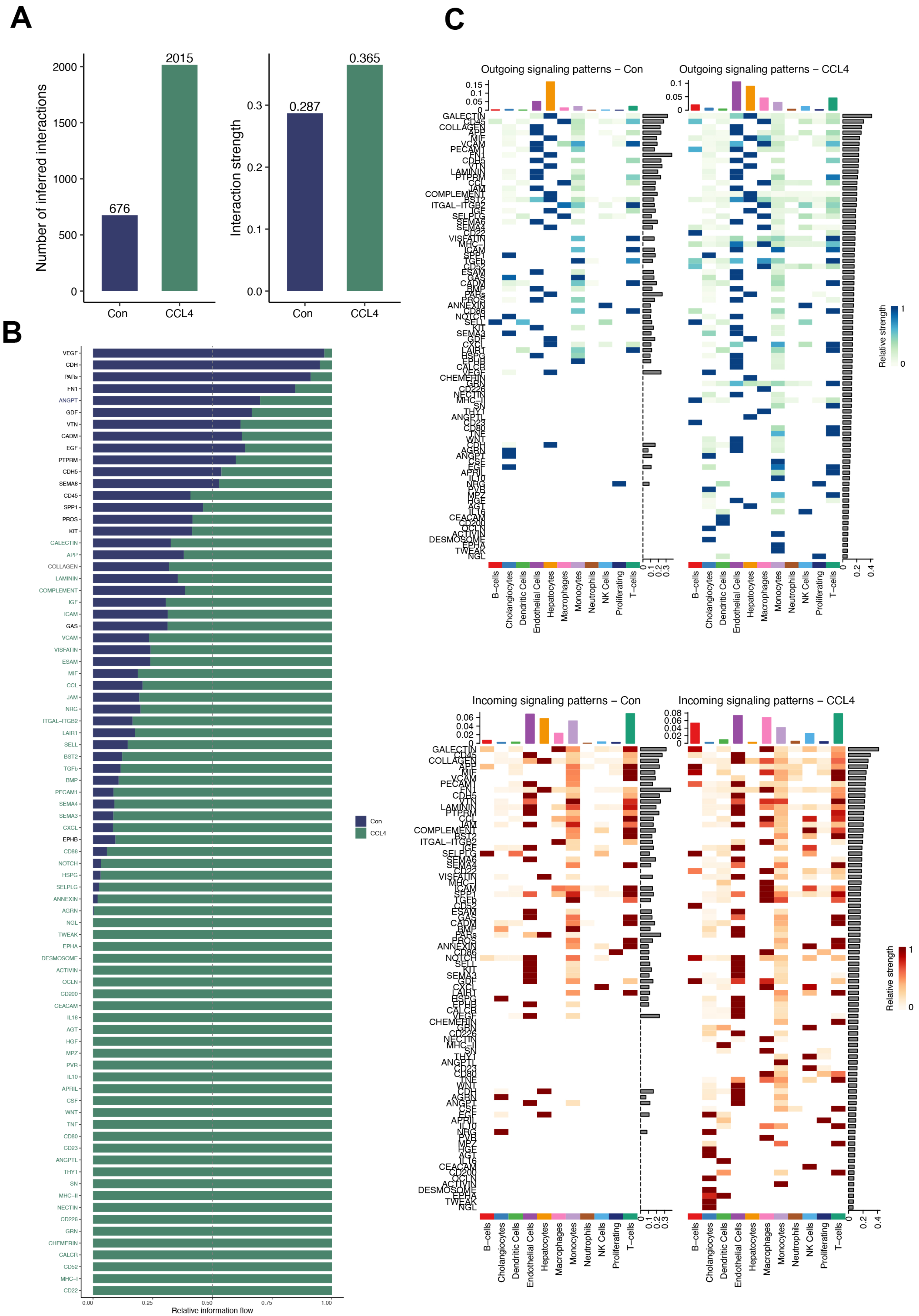

Supplementary Figure 11. Changes in intracellular communications network in the liver niche during CCl4 treatment.
(A) Comparison of the total number of interactions and interaction strength between control and CCL4 treated animals. N=2 Alb-CreERT2 Rosa26^{rbw} mice per group.
(B) Comparison of the signaling pathway enriched in control and CCl4 treated mice based on the relative information flow between pairwise datasets. N=2 Alb-CreERT2 Rosa26^{rbw} mice per group.
(C) Heatmap displaying outgoing and incoming signaling patterns. The scale above the heatmap represents the overall signaling strength in each cell type. The horizontal grey bars to the right of the heatmap depicts the signaling strength of each signaling pathway from all cell type in the liver. The color gradient represents the relative contribution of a cell type to the pathway. N=2 Alb-CreERT2 Rosa26^{rbw} mice per group.

Supplementary Figure 12

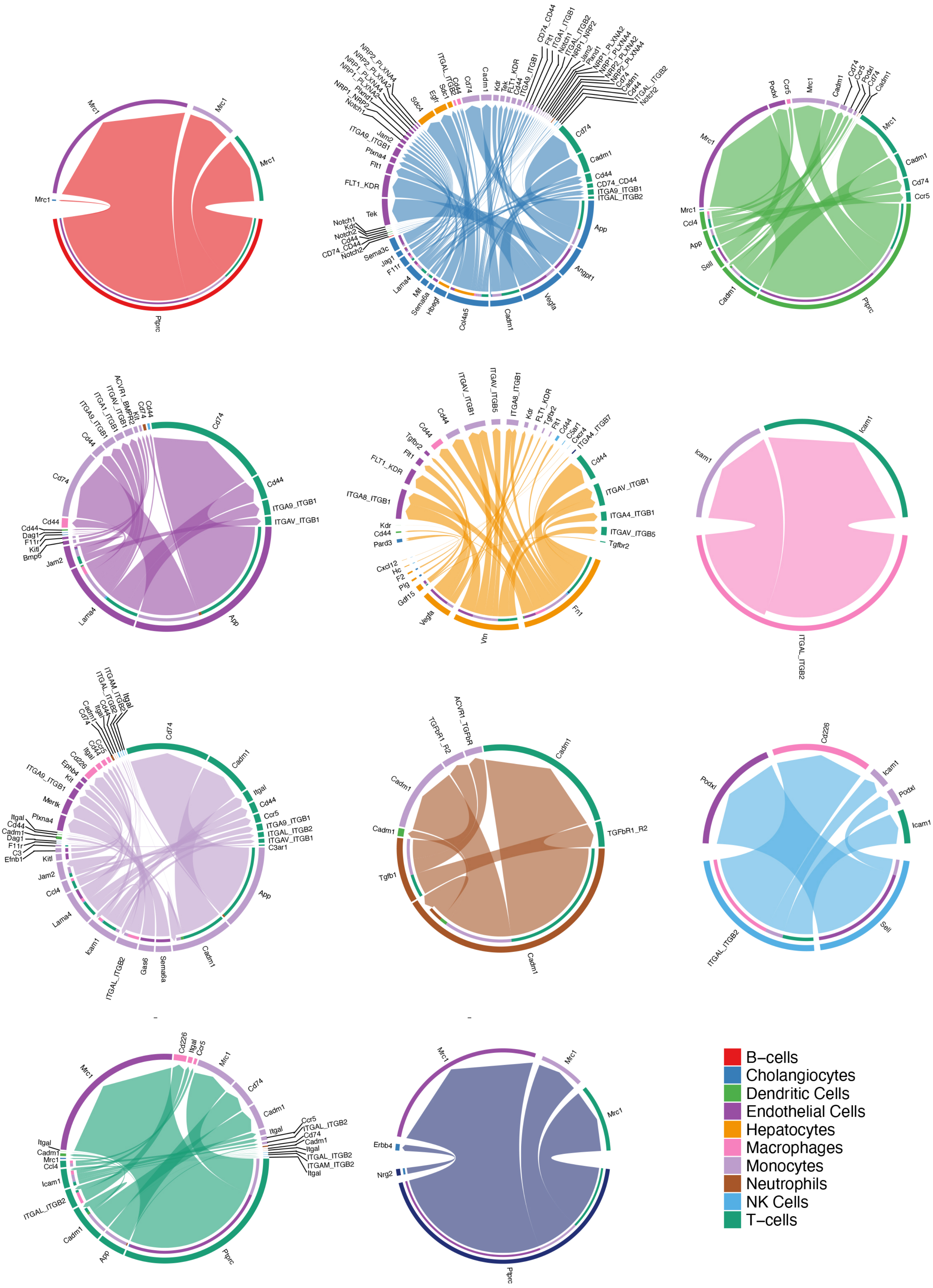

Supplementary Figure 12. Changes in intracellular communications network in the liver niche during CCl4 treatment.

Chord diagram showing downregulated signaling ligand-receptor pairs in CCl4 condition. Each link indicates a ligand-receptor pair. The root of each arrow is the ligand-expressing cell type, and the tip of each arrow is the receiving cell. N=2 Alb-CreERT2 Rosa26^{rbw} mice per group.
