# Supplementary material for: Heterogeneity of hepatocyte dynamics restores liver architecture after chemical, physical or viral damage
Source: Nat Commun. 2024 Feb 10;15:1247. doi: 10.1038/s41467-024-45439-0 (PMC10858916; doi:10.1038/s41467-024-45439-0)
Supplement: Supplementary file 3 — Reporting Summary [file 41467_2024_45439_MOESM3_ESM.pdf]

nature portfolio

Corresponding author(s): Carlos Fernandez-Hernando

Last updated by author(s): Nov 21, 2023

Reporting Summary

Nature Portfolio wishes to improve the reproducibility of the work that we publish. This form provides structure for consistency and transparency in reporting. For further information on Nature Portfolio policies, see our Editorial Policies and the Editorial Policy Checklist.

Statistics

For all statistical analyses, confirm that the following items are present in the figure legend, table legend, main text, or Methods section.

n/a	Confirmed	
☐	☒	The exact sample size (*n*) for each experimental group/condition, given as a discrete number and unit of measurement
☐	☒	A statement on whether measurements were taken from distinct samples or whether the same sample was measured repeatedly
☐	☒	The statistical test(s) used AND whether they are one- or two-sided<br>*Only common tests should be described solely by name; describe more complex techniques in the Methods section.*
☒	☐	A description of all covariates tested
☐	☒	A description of any assumptions or corrections, such as tests of normality and adjustment for multiple comparisons
☐	☒	A full description of the statistical parameters including central tendency (e.g. means) or other basic estimates (e.g. regression coefficient) AND variation (e.g. standard deviation) or associated estimates of uncertainty (e.g. confidence intervals)
☐	☒	For null hypothesis testing, the test statistic (e.g. *F*, *t*, *r*) with confidence intervals, effect sizes, degrees of freedom and *P* value noted<br>*Give P values as exact values whenever suitable.*
☒	☐	For Bayesian analysis, information on the choice of priors and Markov chain Monte Carlo settings
☒	☐	For hierarchical and complex designs, identification of the appropriate level for tests and full reporting of outcomes
☒	☐	Estimates of effect sizes (e.g. Cohen's *d*, Pearson's *r*), indicating how they were calculated

Our web collection on statistics for biologists contains articles on many of the points above.

Software and code

Policy information about availability of computer code

Data collection	Immunofluorescence images were aquired using Nikon microscope (Eclipse 80i upright fluorescent or Eclipse TS100 inverted). Slides were visualized using fluorescent filters for DAPI and the Rainbow colors (mCerulean, mOrange, mCherry and CAG-EGFP). Immunohistochemistry mages were taken with an EVOS microscope.
Data analysis	Graphpad Prism 8, Microsof Excel, Fiji ImageJ-win64, Adobe Photoshop,R package CellChat version 1.6.0, Seurat R package (v3.1.0)

For manuscripts utilizing custom algorithms or software that are central to the research but not yet described in published literature, software must be made available to editors and reviewers. We strongly encourage code deposition in a community repository (e.g. GitHub). See the Nature Portfolio guidelines for submitting code & software for further information.

B

Data

Policy information about availability of data

All manuscripts must include a data availability statement. This statement should provide the following information, where applicable:

- Accession codes, unique identifiers, or web links for publicly available datasets
- A description of any restrictions on data availability
- For clinical datasets or third party data, please ensure that the statement adheres to our policy

Sc-RNA-sequencing data will be deposited in the Gene Expression Omnibus database (GSE222191).

Research involving human participants, their data, or biological material

Policy information about studies with human participants or human data. See also policy information about sex, gender (identity/presentation), and sexual orientation and race, ethnicity and racism.

Reporting on sex and gender	N/A. No human data reported in the study.
Reporting on race, ethnicity, or other socially relevant groupings	N/A. No human data reported in the study.
Population characteristics	N/A. No human data reported in the study.
Recruitment	N/A. No human data reported in the study.
Ethics oversight	N/A. No human data reported in the study.

Note that full information on the approval of the study protocol must also be provided in the manuscript.

Field-specific reporting

Please select the one below that is the best fit for your research. If you are not sure, read the appropriate sections before making your selection.

☒ Life sciences ☐ Behavioural & social sciences ☐ Ecological, evolutionary & environmental sciences

For a reference copy of the document with all sections, see nature.com/documents/nr-reporting-summary-flat.pdf

Life sciences study design

All studies must disclose on these points even when the disclosure is negative.

Sample size	No statistical method was used to predetermine sample size. Samples size was determined in accordance to standard practices in this field of research and based on previous analyses and experience with this or similar experimental design (PMID: 31866223; PMID: 33632818; PMID: 33632817).
Data exclusions	No data points were excluded
Replication	Experiments were replicated as indicated in the figures. Mouse studies included sufficient number to account for biological variability. Multiple techniques and models were used to validate the same finding through different experiments (e.g. scRNAseq, cell-cell interaction analysis, immunofluorescence, immunohistochemistry), each of them including 3-15 biological replicates.
Randomization	All mice in all the experimental groups were matched by age, sex and similar body weight before starting the experiments.
Blinding	The investigators were blinded during the data acquisition in the experiments. Image analysis and quantification was performed in a blinded manner by investigators.

Reporting for specific materials, systems and methods

We require information from authors about some types of materials, experimental systems and methods used in many studies. Here, indicate whether each material, system or method listed is relevant to your study. If you are not sure if a list item applies to your research, read the appropriate section before selecting a response.

Materials & experimental systems

n/a	Involved in the study	
☐	☒	Antibodies
☒	☐	Eukaryotic cell lines
☒	☐	Palaeontology and archaeology
☐	☒	Animals and other organisms
☒	☐	Clinical data
☒	☐	Dual use research of concern
☒	☐	Plants

Methods

n/a	Involved in the study	
☒	☐	ChIP-seq
☒	☐	Flow cytometry
☒	☐	MRI-based neuroimaging

Antibodies

Antibodies used	Post-fixed sections from Alb-CreERT2 Rosa26rbw mice were cut into 6µm-thick sections. Following euthanasia, livers were fixed in 10% formalin (Thermo Fisher Scientific) in Sodium Chloride (NaCl), 0.9% (w/v) Aqueous, Isotonic Saline (RICCA Chemical) overnight at 4 °C. Tissue was then incubated in 15 and 30% sucrose gradients overnight each diluted in 0.9% (w/v) NaCl, embedded in optical cutting temperature compound (Tissue Tek), and stored at −80 °C. Liver lobules were cryosectioned in the transverse axis, and sections were washed with 0.1% Triton X-100 in phosphate-buffered saline (PBS) solution (PBS-T) and immersed in mounting medium with the nuclear fluorescent dye 4',6-Diamidino-2-phenylindole dihydrochloride (DAPI) (Vector laboratories). Slides were immediately visualized using fluorescent filters for DAPI and the Rainbow colors (mCerulean, mOrange, mCherry and CAG-EGFP).<br><br>Immunohistochemistry. Similar to the IF method, livers were fixed in 10% formalin in 0.9% (w/v) NaCl overnight at 4 °C. Tissue was then incubated in 15 and 30% sucrose gradients overnight each diluted in 0.9% (w/v) NaCl, embedded in optical cutting temperature compound (Tissue Tek), and stored at −80 °C. Liver lobules were cryosectioned (6 µm) in the transverse axis and sections were washed twice with PBS during 3 min, incubated with 1% hydrogen peroxidase in PBS for 30 min and blocking with normal goat or donkey serum in PBS for 30 min. After that, liver sections were incubated overnight at 4 °C with the following primary antibodies: Anti-Cytokeratin 19 (Abcam, no. 133496; 1:100), anti-Glutamine Synthetase (Abcam, no. 197024; 1:50), anti-CD31 (Abcam, no. 28364; 1:100), anti-CD68 (Abcam, no. 125047; 1:200), anti-Ki67 (Abcam, no. 15580; 1:100) and anti-CD45 (Novus Biologicals no. AF114; 1:100). The following day, sections were washed with 3 times with PBS for 5 min and incubated 30 min at room temperature with the biotinylated secondary antibodies (Rockland, anti-rabbit no. 611-106-122 and anti-goat no. 605-4613; 1:500 in PBS). Then, slides were washed 3 times with PBS for 2 min and incubated at room temperature with streptavidin peroxidase conjugated (Rockland, no. S000-03) for 30 min at 1:500 in PBS. Finally, sections were incubated with 3,3'-Diaminobenzidine (DAB) Substrate (Rockland, no. DAB-10) for 3-8 min and counterstained with hematoxylin (Millipore Sigma). For apoptosis staining, we used the TUNEL Assay Kit- HRP-DAB (Abcam, no. 206386).
Validation	Antibodies and staining have been previously validated and extensively used in the field for similar staining.

Animals and other research organisms

Policy information about studies involving animals; ARRIVE guidelines recommended for reporting animal research, and Sex and Gender in Research

Laboratory animals	Homozygous Rosa 26-Rainbow Cre-mediated recombination mice (Rosa26rbw) 17 were obtained from Prof. Daniel Greif at Yale School of Medicine and previously generated by Prof. Irv Weissman at Stanford University. These mice were crossed with homozygous TMX-inducible albumin Cre mice (Alb-CreERT2) provided by Prof. Daniel Metzger to generate heterozygous Alb CreERT2 Rosa26rbw mice, which specifically expressed in the liver the rainbow colors after TMX injection. These mice were subjected of the liver damage models used in our study. Agarose gel electrophoresis was performed to confirm the genotype of Rosa26rbw, and Alb-CreERT2 mice. All the experiments were approved by the Institutional Animal Care Use Committee of Yale University School of Medicine. Mice used in all experiments were sex and age matched and kept in individually ventilated cages in a pathogen-free facility.
Wild animals	No wild animals were used in this study
Reporting on sex	Findings reported in the manuscript apply to both male and female mice as mice sex is indicated in each experiment.
Field-collected samples	This study did not involve field-collected samples
Ethics oversight	All procedures were approved by the Institutional Animal Care and Use Committee (IACUC - Yale University)

Note that full information on the approval of the study protocol must also be provided in the manuscript.
